# Flutamide Alters the Expression of Chemerin, Apelin, and Vaspin and Their Respective Receptors in the Testes of Adult Rats

**DOI:** 10.3390/ijms21124439

**Published:** 2020-06-22

**Authors:** Malgorzata Brzoskwinia, Laura Pardyak, Agnieszka Rak, Alicja Kaminska, Anna Hejmej, Sylwia Marek, Malgorzata Kotula-Balak, Barbara Bilinska

**Affiliations:** 1 Department of Endocrinology, Institute of Zoology and Biomedical Research, Faculty of Biology, Jagiellonian University, 30-387 Krakow, Poland; m.brzoskwinia@doctoral.uj.edu.pl (M.B.); laura.pardyak@doctoral.uj.edu.pl (L.P.); ala.kaminska@doctoral.uj.edu.pl (A.K.); anna.hejmej@uj.edu.pl (A.H.); s.marek@doctoral.uj.edu.pl (S.M.); 2 Department of Physiology and Toxicology of Reproduction, Institute of Zoology and Biomedical Research, Faculty of Biology, Jagiellonian University, 30-387 Krakow, Poland; agnieszka.rak@uj.edu.pl; 3 University Centre of Veterinary Medicine, University of Agriculture in Krakow, 30-059 Krakow, Poland; malgorzata.kotula-balak@urk.edu.pl

**Keywords:** chemerin, apelin, vaspin, adipokine receptors, testes, Leydig cells, flutamide, rat

## Abstract

Adipokines influence energy metabolism and have effects on male reproduction, including spermatogenesis and/or Sertoli cell maturation; however, the relationship between these active proteins and androgens in testicular cells is limited. Here, we studied the impact of short-term exposure to flutamide (an anti-androgen that blocks androgen receptors) on the expression of chemerin, apelin, vaspin and their receptors (CCRL2, CMKLR1, GPR1, APLNR, GRP78, respectively) in adult rat testes. Moreover, the levels of expression of lipid metabolism-modulating proteins (PLIN1, perilipin1; TSPO, translocator protein) and intercellular adherens junction proteins (nectin-2 and afadin) were determined in testicular cells. Plasma levels of adipokines, testosterone and cholesterol were also evaluated. Gene expression techniques used included the quantitative real-time polymerase chain reaction (qRT-PCR), Western blot (WB) and immunohistochemistry (IHC). The androgen-mediated effects observed post-flutamide treatment were found at the gonadal level as chemerin, apelin, and vaspin gene expression alterations at mRNA and protein levels were detected, whereas the cellular targets for these adipokines were recognised by localisation of respective receptors in testicular cells. Plasma concentrations of all adipokines were unchanged, whereas plasma cholesterol content and testosterone level increased after flutamide exposure. Differential distribution of adipokine receptors indicates potential para- or autocrine action of the adipokines within the rat testes. Additionally, changes in the expression of PLIN1 and TSPO, involved in the initial step of testosterone synthesis in Leydig cells, suggest that testicular cells represent a target of flutamide action. Increase in the gene expression of PLIN1 and TSPO and higher total plasma cholesterol content indicates enhanced availability of cholesterol in Leydig cells as a result of androgen-mediated effects of flutamide. Alterations in adherens junction protein expression in the testis confirm the flutamide efficacy in disruption of androgen signalling and presumably lead to impaired para- and autocrine communication, important for proper functioning of adipokines.

## 1. Introduction

Adipose-derived hormones, including adiponectin, leptin, visfatin, resistin, chemerin, apelin, vaspin and many others, are called adipokines. These bioactive proteins act via binding to specific receptors that are frequently located on cells that do not produce adipokines, suggesting a regulatory mechanism that functions through endo- and/or paracrine interactions [1]. Adipokines, besides influencing glucose utilisation, insulin sensitivity and energy homeostasis, have effects on both female and male reproduction [2,3,4]. The effects of leptin on reproduction and fertility have been investigated [5,6] and its impact to inducing puberty has been reviewed [7]. It was demonstrated that adiponectin, along with other adipokines (e.g., leptin, resistin), plays a role in the interaction between metabolism and reproductive functions at the hypothalamic, pituitary and gonadal levels (for review, see [8,9]) in humans, rodents, and agronomic species. It should be added that a gender-dependent function of adipokines (e.g., vaspin) has been recognised in humans, as women have markedly increased plasma concentration of vaspin relative to men [10]. Deregulation of adipokine levels may lead to various effects, e.g., obesity, type 2 diabetes and, in consequence, hypogonadism and subfertility [11,12]. Obesity, as disruptor of the male fertility, has been shown to be associated with disturbed sperm motility and decreased testosterone levels [13]. Interestingly, a recent meta-analysis indicated that resistin and visfatin affect spermatogenesis [14]. In a very recent study it was demonstrated that long treatment of human cultured Sertoli cells with chemerin, visfatin or resistin at high concentrations (which are often observed in obese men) suppresses FSH receptor expression and up-regulates that of the cytochrome P450 CYP26A1, which in turn induces a phenotype characteristic of the pre-pubertal state [15]. It was suggested that these adipokines negatively affect Sertoli cell maturation, possibly contributing to testis dysfunction and fertility perturbations associated with obesity. Although the presence of chemerin and visfatin has been detected in the human testis, data concerning the pathogenic role of other adipokines in male reproductive disorders remain to be investigated.

It is to the point here that the link between adipokines and sex hormones is much better understood in females than in males; however, the two most studied adipokines, leptin and adiponectin, have emerged recently as pivotal signals involved in male reproduction [16,17,18]. It was reported that in rat testes leptin through its own receptor inhibits testosterone secretion and leads to the impairment of steroidogenic function of Leydig cells [19,20]. Adiponectin and its receptors have been demonstrated in the chicken testis and found to influence steroidogenesis and spermatogenesis [21]. A relationship between testosterone and adiponectin has been reported in the rat, showing that a developmental exposure to isoflavones increases serum adiponectin level and decreases serum testosterone level [22]. A role of adiponectin as a factor modulating testis functioning during aging in mice has recently been investigated [23]. It has also been suggested that in rodent and chicken testes visfatin influences testicular aging by causing decreases in serum testosterone concentrations [24,25]. Another adipokine, resistin, has been shown to increase testosterone synthesis, and perhaps Leydig cell proliferation [26]. Similarly, Hameed and colleagues [27] have reported increased testosterone levels in rat Leydig cells in vitro after administration of visfatin. Visfatin has been observed in human spermatozoa and concentrations in seminal plasma were found to be higher than blood plasma [28]. Lastly, a link between adiponectin and leptin and estrogen signalling interactions has been reported in Leydig cell tumours [29]. Since data regarding apelin, chemerin and vaspin functioning at the testicular level are limited, or even completely absent [4,30,31], understanding the relationship between these adipokines and androgens is of special interest. Precise cellular localisation of apelin, chemerin and vaspin receptors is important to show the adipokine cellular targets within the testis.

Androgens play a vital role in the regulation of reproduction in males. Their effects are mediated by the androgen receptor (AR). In the testis, the AR is present in testicular Leydig, Sertoli, and peritubular cells [32]. This ligand-induced nuclear receptor plays a central role in androgen signalling. To test whether the disruption of androgen signalling may have an effect on the presence of adipokines within the testes, we used a pure nonsteroidal anti-androgen, flutamide, which is classified as a nuclear AR antagonist that inhibits androgen by blocking its binding to the AR [33,34]. It was demonstrated that flutamide inhibits androgen action by blocking AR transcriptional activity, rather than by affecting AR nuclear localisation in target cells [35]. In human medicine, flutamide is commonly used to treat benign hyperplasia and carcinoma of the prostate. In animal models, the long-term effects of this anti-androgen on male reproduction are well characterised [36,37,38], although its short-term effects on the male gonad are still elusive [39]. We previously showed that flutamide produced diverse effects on different cellular targets within adult rat testes. Short-term flutamide treatment had no effect on the morphology of the seminiferous epithelium; however, it caused an apparent enlargement of the interstitial tissue in which Leydig cells are located [40]. In the light of these findings, we proposed that the tubular compartment and the intertubular compartment are variously sensitive to androgens and a different set of cellular factors may be stimulated (or inhibited) by flutamide in various cell types. In this study we asked whether short-term androgen withdrawal may induce changes in the expression of adipokines and their receptors in Leydig cells within adult rat testes. We aimed to investigate the mRNA and protein expression patterns of (1) chemerin and its three receptors, G protein coupled receptor 1 (GPR1), chemokine like receptor 1 (CMKLR1) and chemokine CC motif receptor-like 2 (CCRL2); (2) apelin and its specific receptor APLNR; and (3) vaspin and the glucose-regulated protein 78 (GRP78) in the testes of control and flutamide-treated rats using qRT-PCR, Western blot (WB), and immunohistochemistry (IHC). Plasma levels of chemerin, apelin and vaspin were also measured. Additionally, expression of two proteins (potential markers of lipid metabolism variations in Leydig cells) including perilipin1 (PLIN1) and the translocator protein (TSPO) were detected to reveal functional alterations in Leydig cells [41,42]. PLIN1 coats lipid droplets and serves as a protector of energy stores by blocking lipid hydrolysis, while TSPO is localised in the outer mitochondrial membrane of Leydig cells and is involved in lipid homeostasis [41,42]. As cholesterol availability is important for testosterone synthesis in Leydig cells [43], plasma cholesterol content was also measured.

It has been proposed that proteins that constitute intercellular junctions within seminiferous tubules may provide an “early warning” for testicular toxicants [44]. In this context, we asked whether flutamide alters the expression of adherens junction proteins, nectin-2 and afadin, which are present in Leydig cells. If it is the case, effectiveness of androgen deprivation by flutamide will be confirmed. We hypothesise that androgen signalling disrupted by flutamide may have an impact on the expression of chemerin, apelin and vaspin, and that altered expression of junctional proteins induced by this antiandrogen may lead to impaired para- and autocrine communication, important for proper functioning of adipokines. It is also tempting to speculate that the adipokines acting through their respective receptors may contribute to modulation of Leydig cell functioning. The above working hypotheses sound reasonable because the synthesis and secretion of adipokines within the testis are likely to be controlled by androgen-mediated local interactions and can be working in concert with junctional proteins.

## 2. Results

As has been stated in the introduction, short-term flutamide exposure influences morpho-functional aspects of spermatogenesis in the rat [40,45]. On the other hand, it has been shown that AR-mediated androgen signalling plays an important role in male metabolism by affecting the energy balance [46]. Androgens are implicated in energy homeostasis, i.a., regulating cholesterol synthesis and lipid metabolism in the liver [47]. Here, we focus our attention on the influence of androgen signalling disrupted by flutamide on the expression of chemerin, apelin and vaspin, as well as on the expression of lipid metabolism-modulating proteins (PLIN1 and TSPO) at the gonadal level. A possible contribution of these adipokines in the modulation of Leydig cell functioning, especially cholesterol availability, is also taken into consideration. Of note, to show the adipokine cellular targets within the testes, localisation of the chemerin, apelin and vaspin receptors is analysed immunohistochemically. Dependent on the receptor distribution, para- and/or autocrine action of the abovementioned adipokines is suggested. Finally, to confirm the flutamide effectiveness in the disruption of androgen signalling within the testis, adherens junction protein expression is determined, while serum concentration of testosterone is measured to confirm an impairment of the negative feedback at the level of the pituitary gland. It is suggested that post-flutamide changes in junctional protein expression may disturb para- and autocrine communication, crucial for adipokines’ functioning.

### 2.1. Expression of Adipokines and Their Receptor Genes in Rat Testes, Effect of Flutamide

#### 2.1.1. Immunolocalisation of Adipokines and Their Receptors

**Figure 1:**
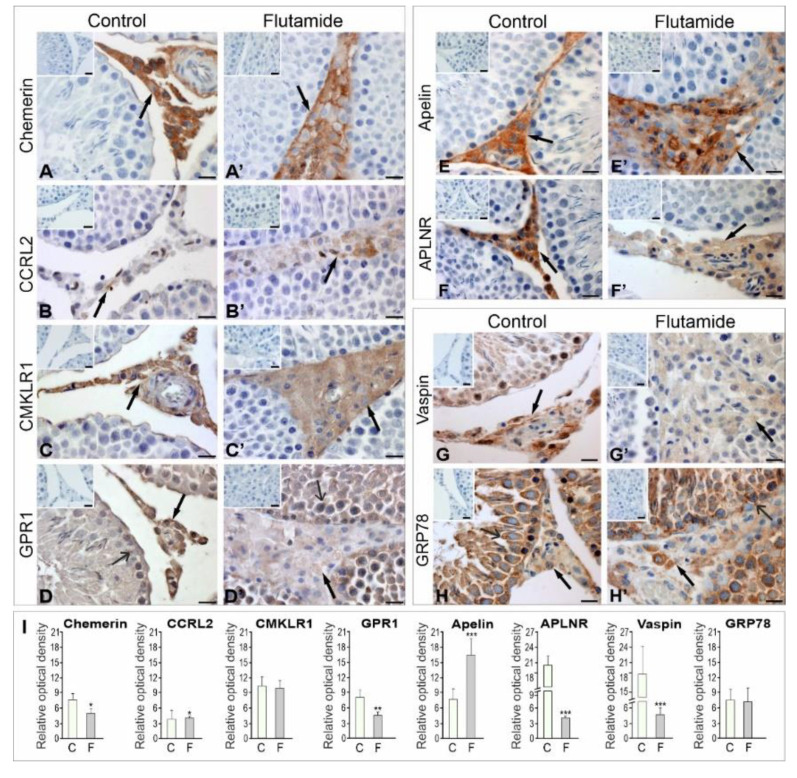
Qualitative (**A**–**H**, **A’**–**H’**) and quantitative (**I**) analyses of immunohistochemical staining of chemerin (**A**,**A’**), CCRL2 (**B**,**B’**), CMKLR1 (**C**,**C’**), GPR1 (**D**,**D’**), apelin (**E**,**E’**), APLNR (**F**,**F’**), vaspin **(G**,**G’)** and GRP78 **(H**,**H’)**. Representative microphotographs of control **(A**–**H)** and flutamide-treated testes (**A’**–**H’**). Additionally stained with Mayer’s haematoxylin. Bars = 10 μm. Positive staining for chemerin, CCRL2, CMKLR1, apelin and APLNR are restricted to Leydig cells (arrows), whereas staining of GPR1, vaspin and GRP78 is visible in Leydig cells (arrows) and seminiferous tubules (open arrows). Note the decreased levels of chemerin (**A’**), GPR1 (**D’**), APLNR (**F’**) and vaspin (**G’**) and increased levels of CCRL2 (**B’**) and apelin (**E’**) intensities after flutamide administration. The intensity of CMKLR1 and GRP78 staining in flutamide (**C’,H’**) and control (**C**,**H**) Leydig cells are similar. No immunopositive staining of chemerin, apelin and vaspin and their receptors was observed when the primary antibodies are omitted (**A**–**H**,**A’**–**H’,** insertion in upper left corner of microphotographs). Histograms (**I**) depicting staining intensities of chemerin, apelin, vaspin, and their receptors are expressed as relative optical density of the brown staining product in Leydig cells. The results are presented as means ± SD. Statistically significant differences are flagged with asterisks (* *p* < 0.05, ** *p* < 0.01, *** *p* < 0.001). Control (*n* = 6) and flutamide-treated (*n* = 6) animals; C: control, F: flutamide.

Positive signals for adipokines and their receptors were present in the testes of all rats assessed as shown using IHC (Figure 1**)**. Immunoreactive signals for chemerin and its two receptors (CCRL2 and CMKLR1) (Figure 1A–C) and apelin and its receptor APLNR (Figure 1E,F) were confined to Leydig cells, whereas GPR1 signals of moderate intensity were observed in Leydig cells and seminiferous tubules (Figure 1D). Vaspin and GRP78 signals were distributed throughout both testicular compartments and were from moderate to strong intensity in Leydig cells, Sertoli cells and germ cells (Figure 1G,H), indicating potential para- or autocrine action of the adipokines within the rat testes. Flutamide treatment resulted in decreased chemerin, CMKLR1, GPR1 and APLNR signals (Figure 1A’–B’,D’,F’), while increases in apelin (Figure 1E’) were observed in Leydig cells relative to the control cells (Figure 1A,B, D–F). The CCRL2 signals were very weak in Leydig cells of flutamide-exposed testes (Figure 1C’) and almost invisible in the respective control testes (Figure 1C). After flutamide exposure, vaspin signals decreased, while the intensity of GRP78 remained strong within the seminiferous tubules and was weak to moderate within the interstitium (Figure 1G’–H’). No signals were detected for chemerin, apelin and vaspin or their receptors when primary antibodies were omitted and replaced by normal horse or goat serum (Figure 1A–H, A’–H’, insertion in upper left corner of microphotographs).

Qualitative analyses of signals from adipokines and their receptors were confirmed using quantitative image analysis. Statistically significant differences from control values are denoted as * *p* < 0.05; ** *p* < 0.01; *** *p* < 0.001 (Figure 1I).

#### 2.1.2. Expression Levels of Adipokine and Adipokine Receptor Proteins

**Figure 2:**
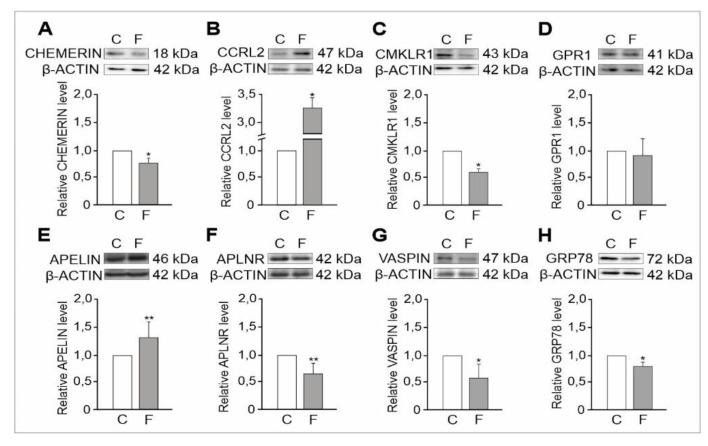
Representative Western blots assessing the relative expression of chemerin (**A**), CCRL2 (**B**), CMKLR1 (**C**), GPR1 (**D**)**,** apelin (**E**), APLNR **(F),** vaspin (**G**) and GRP78 (**H**) proteins in control and flutamide-treated testes. Normalisation was performed with β-Actin as a loading control. Protein levels within control testes were given a value of 1. Data obtained from three separate analyses are presented as means ± SD. Statistically significant differences are flagged with asterisks (* *p* < 0.05, ** *p* < 0.01) between control (*n* = 6) and flutamide-treated (*n* = 6) animals; C: control, F: flutamide.

Proteins were observed as single bands near 18 kDa (chemerin), 47 kDa (CCRL2), 43 kDa (CMKLR1), 41 kDa (GPR1), 46 kDa (apelin), 42 kDa (APLNR), 47 kDa (vaspin), 72 kDa (GRP78) and 42 kDa (β-Actin) (Figure 2A–H and Supplementary Figure S1). Quantitative measurements of protein band intensities indicated changes in the expression levels in testes homogenates of flutamide-treated vs. control rats. Chemerin and CMKLR1 levels decreased (*p* < 0.05) (Figure 2A,C), and CCRL2 levels increased relative to controls (*p* < 0.05) (Figure 2B). Further, no significant change in levels of GPR1 (Figure 2D) were detected compared to its respective controls (Figure 2A–D). Increased levels of apelin (*p* < 0.01) and decreased levels of APLNR (*p* < 0.01), vaspin, and GRP78 (*p* < 0.05) vs. controls were observed (Figure 2E–H).

#### 2.1.3. mRNA Expression Levels of Adipokines and Their Receptors

**Figure 3:**
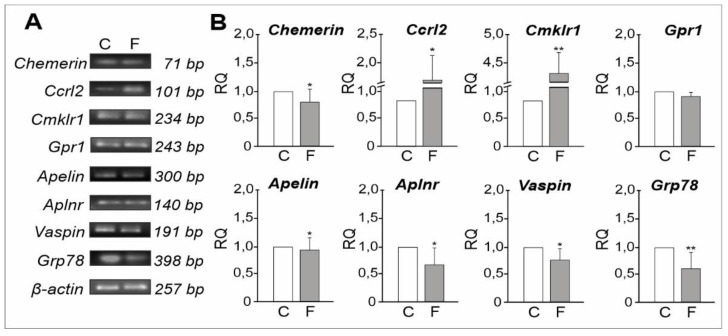
Results of RT-PCR and qRT-PCR in control and flutamide-treated testes. The levels of *Chemerin*, *Ccrl2*, *Cmklr1*, *Gpr1*, *Apelin*, *Aplnr*, *Vaspin*, and *Grp78* mRNAs were expressed by electrophoresis bands (**A**). Relative expression levels of respective mRNAs (RQ) were determined using qRT-PCR. (**B**) As reference gene, the *β-actin* mRNA level was shown. Relative expression levels of genes (RQ) were normalised to the mean expression of *β-actin*, *Gapdh* and *Rpl13a* as an internal control. Relative quantification (RQ) is expressed as means ± SD. Statistically significant differences are flagged with asterisks (* *p* < 0.05, ** *p* < 0.01); control (*n* = 6) and flutamide-treated (*n* = 6) animals; C: control, F: flutamide.

Electrophoresis revealed PCR-amplified products associated with chemerin, apelin, vaspin and their receptors (Figure 3A). Real-time PCR assessing mRNA expression levels revealed the down-regulation of *Chemerin* (*p* < 0.05), while transcripts of two chemerin receptors (*Ccrl2 and Cmklr1*) were up-regulated (*p* < 0.05, *p* < 0.01, respectively), and no significant changes in *Gpr1* expression levels were noted post-flutamide treatment relative to controls (Figure 3B). *Apelin, Aplnr, Vaspin,* and *Grp78* mRNA expression levels were down-regulated (*p* < 0.05 and *p* < 0.01) following flutamide exposure compared to levels of controls (Figure 3B).

Changes in immunoreactivity associated with protein levels of adipokines and their receptors and results regarding mRNA expression levels suggest a direct impact of flutamide at the gonadal level.

### 2.2. Plasma Concentrations of Adipokines, Effect of Flutamide

No statistically significant differences were noted in plasma levels of chemerin, apelin and vaspin in control and flutamide-treated males. (Table 1). It seems likely that total adipokine concentrations are not affected by the anti-androgen treatment.

### 2.3. Testosterone Blood Plasma Concentration, Effects of Flutamide

Plasma testosterone concentrations markedly (6-fold) increased in flutamide-treated samples (21.080 ± 4.879 ng/mL) relative to control samples (3.520 ± 0.901 ng/mL; *p* < 0.01). This indicates the presence of flutamide-induced effects within the hypothalamus–pituitary–gonadal axis.

### 2.4. Plasma Cholesterol Content, Effects of Flutamide

Plasma cholesterol content (0.741 mmol/mL ± 0.019) was elevated in flutamide-exposed rats when compared to the controls (0.593 mmol/mL ± 0.031, *p* < 0.01), which may suggest that flutamide altered lipid homeostasis.

### 2.5. Expression of PLIN1 and TSPO Genes in Rat Testes, Effect of Flutamide

**Figure 4:**
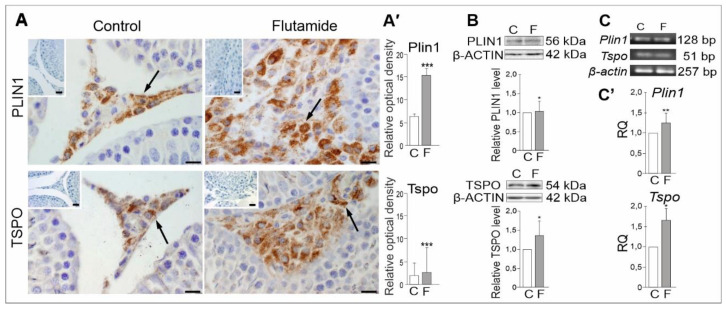
Immunohistochemical localisation of PLIN1 and TSPO (**A**) and densitometric image analysis (**A’**), protein (**B**) and mRNA (**C**, **C’**) expression levels in control and flutamide-treated testes. Additionally stained with Mayer’s haematoxylin. Bars = 20 μm. In (**A**)**,** representative microphotographs showing the cellular localisation of PLIN1 and TSPO signals in Leydig cells (arrows). Note higher intensity staining for both proteins in Leydig cells following flutamide exposure as shown also in (**A’**) Representative Western blots (**B**) and the relative levels of PLIN1 and TSPO proteins were normalised using β-Actin, which served as an internal protein loading control. Protein levels within control testes were given a value of 1. Representative RT-PCR and qRT-PCR analyses of *Plin1* and *Tspo* mRNA expression levels in control and flutamide-exposed testes are shown in **C** and **C’**, respectively. Relative quantification (RQ) is expressed as mean ± SD. Statistically significant differences are flagged with asterisks (* *p* < 0.05, ** *p* < 0.01, *** *p* < 0.001) between control (*n* = 6) and flutamide-treated animals (*n* = 6); C: control, F: flutamide.

Immunohistochemistry analyses revealed positive staining for PLIN1 and TSPO throughout the interstitial area (Figure 4A). Following flutamide treatment, both PLIN1 and TSPO signals were confined to Leydig cells and increased in intensity in comparison to the respective controls. Qualitative IHC was confirmed by densitometric image analysis (Figure 4A’). Concomitantly, significant increase in PLIN1 and TSPO protein levels were observed in flutamide vs. control rats (*p* < 0.05), and corresponding mRNAs were also up-regulated (*p* < 0.01; *p* < 0.05, respectively) according to WB and qRT-PCR analyses, respectively (Figure 4B,C’ and Supplementary Figure S1). Proteins were observed as single bands near 56 kDa (PLIN1), 54 kDa (TSPO) and 42 kDa (β-Actin) (Figure 4B), while electrophoresis revealed PCR-amplified products corresponded to expected sizes of 128, 51 and 257 bp for *Plin1*, *Tspo*, and *β-actin*, respectively (Figure 4C and Supplementary Figure S1). Alterations in *Plin1* and *Tspo* gene expression levels indicated that flutamide treatment may have an impact on cholesterol availability in Leydig cells and suggest the impact of flutamide at the gonadal level.

### 2.6. Expression of Nectin2 and Afadin Genes in Rat Testes, Effect of Flutamide

**Figure 5:**
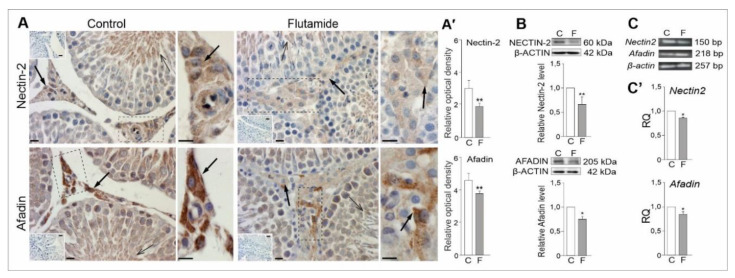
Immunohistochemical localisation of nectin-2 and afadin (**A**) and densitometric image analysis (**A’**), along with protein (**B**), and mRNA (**C**,**C’**) expression levels in testes of control and flutamide-exposed rats. Additionally stained with Mayer’s haematoxylin. Frames indicate the location of the higher-magnification views. Bars = 20 μm. In (**A**), representative microphotographs showing the cellular localisation of nectin-2 and afadin signals in Leydig cells (arrows) and in the apical compartment of seminiferous tubule (open arrows) of control and flutamide-treated testes.Note lower intensity staining for both proteins in Leydig cells following flutamide exposure as shown also in (**A’**). For details, see the higher magnification insets. Representative Western blots are shown in (**B**)**,** and the relative levels of nectin-2 and afadin proteins were normalised using the internal protein loading control, β-Actin. Protein levels within control testes were given a value of 1. Representative RT-PCR and qRT-PCR analyses of *Nectin2* and *Afadin* mRNA expression levels in the testes of control and flutamide-exposed males are shown in (**C**,**C’**), respectively. Relative quantification (RQ) is expressed as mean ± SD. Statistically significant differences are flagged with asterisks (* *p* < 0.05, ** *p* < 0.01) between control (*n* = 6) and flutamide-treated (*n* = 6) animals; C: control, F: flutamide.

Specific post-flutamide effects in testes, determined via IHC, WB and qRT-PCR (Figure 5) were as follows: reduced staining intensities of nectin-2 and afadin in Leydig cells (Figure 5A), confirmed by densitometric image analysis (Figure 5A’), significant decreases in levels of both proteins (*p* < 0.01; *p* < 0.05) (Figure 5B and Supplementary Figure S1) and the down-regulation of nectin-2 and afadin mRNAs in testes of flutamide-treated rats (*p* < 0.05) compared to those of controls (Figure 5C’). Proteins were observed as single bands near 60 kDa (nectin-2), 205 kDa (afadin) and 42 kDa (β-Actin) (Figure 5B), whereas electrophoresis revealed that PCR products matched with the expected sizes of 150, 218 and 257 bp for *Nectin2, Afadin*, and *β-actin*, respectively (Figure 5C and Supplementary Figure S1). Alterations in *Nectin2* and *Afadin* gene expression levels indicated that the short-term flutamide treatment is sufficient to induce specific effects on cell-cell junction proteins within Leydig cells.

## 3. Discussion

Although putative roles for adiponectin and leptin as endocrine factors that influence male reproduction in health and disease (in humans and laboratory animals) have been highlighted recently [18,48,49], limited information regarding the functioning of other adipokines within the male gonad is available. In this study, we sought to characterise the effects of short-term flutamide treatment on the expression of chemerin, apelin and vaspin and their receptors in adult rat testes. First, we showed reduced staining intensities of chemerin and CMKLR1 in Leydig cells and decreased levels of both proteins and chemerin mRNA, but increased CMKLR1 mRNA expression in flutamide-treated testes. On the other hand, although CCRL2 mRNA and protein levels were up-regulated after flutamide exposure, a role for CMKLR1, rather than CCRL2, in mediating chemerin functioning in Leydig cells is suggested since the immunoreactive signal for CCRL2 was very weak in these cells. Of note, CCRL2 receptor has earlier been shown as a non-signalling receptor for chemerin in the ovary [50]. It seems likely that up- or down-regulation of protein expression post-flutamide exposure indicates an altered functional activity of Leydig cells which involves altered autocrine interactions. To date, only Li and colleagues [51,52] have shown the existence of chemerin and its receptors in Leydig cells, which has suggested a role for chemerin as a peripherally acting molecule in the testes. The research also suggested a suppressive effect for chemerin on hCG-stimulated testosterone synthesis in cultured rat Leydig cells. A very recent study involving the use of CMKLR1 knockout mice (CMKLR1-/-) assessed plasma testosterone levels and revealed that expression of enzymes implicated in testosterone production was lower in knockout mice compared to wild-type animals [53]. This work highlighted the importance of chemerin, which functions through CMKLR1 in male steroidogenesis. Herein, we demonstrated that GPR1 mRNA and protein levels of the third chemerin receptor in flutamide-treated and control animals were slightly different. GPR1 immunoexpression was decreased in Leydig cells and remained unchanged in seminiferous tubules of flutamide-treated vs. control rats. This may suggest a dependence of chemerin on AR signalling through GPR1, which is specific to Leydig cells, rather than seminiferous tubules. The results indicated that Leydig cells are the location where chemerin operates through its own receptors, which is consistent with previously published reports [21,54,55] showing adiponectin and its receptors present in rat and chicken testicular Leydig cells.

Further, we showed that apelin and APLNR were distributed throughout Leydig cells, but were not present in other cell types, indicating its autocrine mode of action within the Leydig cells. Flutamide led to apparent decreases in APLNR signal intensities in Leydig cells; however, the immunoreactivity of apelin increased. Such findings may be a result of the reduced activity of apelin at the gonadal level after ARs were blocked by flutamide in adult rats. A suppressive role of apelin on central hormonal regulation has been described by Sandal et al. [31], who noted that a diminished number of Leydig cells and reduced serum testosterone concentrations occurred after intracerebroventricular apelin-13 infusion. Although treatment with flutamide significantly down-regulated mRNA and protein expression levels of chemerin, no correlation between mRNA and protein levels of apelin or CMKLR1 (as indicated above) in the testes of flutamide-treated rats was found. We speculate that decreases in apelin mRNA levels and concomitant increases in protein levels may suggest that enhanced translation of the protein occurs in testes of treated males. On the other hand, increases in CMKLR1 mRNA and decreases in its protein expression may reflect that increased degradation of the protein occurred as a result of flutamide exposure. According to Greenbaum et al. [56], both measurements of mRNA and protein levels are important for understanding cellular functioning, but protein levels cannot be predicted solely from mRNA levels.

Our results provide new information indicating that interstitial tissue is the only place within the rat testis in which chemerin and apelin may act through CCRL2, CMKLR1 and APLNR, respectively. Conversely, the vaspin signals were localised to Leydig cells, whereas the vaspin receptor (GRP78) was present in both testicular compartments. Wide allocation of GRP78 within the seminiferous epithelium suggests a possible role for vaspin in the regulation of Sertoli and germ cell functioning, rather than its involvement in the modulation of Leydig cell functioning. It is likely that vaspin acts locally in the seminiferous tubular compartment as a paracrine and/or autocrine factor, presumably influencing spermatogenesis. On the other hand, the unchanged expression pattern of GRP78 in the seminiferous tubules of flutamide-treated rats indicates that in contrast to its anti-androgenic effects on vaspin, GRP78 expression was not affected by the anti-androgen. The reason disturbed androgen signalling (after treatment with flutamide) differentially affects expression of vaspin and GRP78 in various cellular targets remains unclear; however, results may suggest that the deregulation of paracrine action occurs in both testicular compartments of the rat. It is to the point here that the plasma chemerin, apelin and vaspin levels remained unchanged post-flutamide treatment, indicating no androgen-mediated effects of flutamide on the circulating adipokines’ concentrations. However, higher serum chemerin, apelin and vaspin levels have earlier been reported in women with polycystic ovary syndrome and hyperandrogenemia [4,57].

In addition to flutamide-induced effects at the hypothalamus–pituitary level reported both previously [58,59] and in the present study (6-fold increase in plasma testosterone concentration), functional alterations at the gonadal level, specifically in Leydig cells, were demonstrated. In the enlarged interstitial space of flutamide-treated testes, PLIN1 and TSPO signals were strong and the expression of both mRNA and protein levels was significantly elevated. Of note, cholesterol is a substrate for testosterone production and is received from several cellular sources including lipid droplets, which are located in Leydig cells [60]. Accordingly, PLIN1 proteins are widely distributed in lipid droplets and in the endoplasmic reticulum of adipocytes, suggesting that perilipin levels are influenced by nutritional changes [61]. In a clinical report previously published, the relationship between plasma cholesterol and apelin levels were reported in patients with hypercholesterolemia for whom lower apelin levels were determined [62]. Similarly, a relationship between high quantities of lipids in the diet of mothers and expression of adiponectin and leptin in testes of male rat offspring was detected [63,64]. Based on increased plasma cholesterol content and increased PLIN1 and TSPO signals after flutamide exposure, it is suggested that availability of cholesterol in Leydig cells is enhanced. Recently, Milon et al. reported [65] lipid homeostasis and metabolism to be affected by endogenous and/or exogenous estrogens. However, the idea that adipokines acting through their own receptors may increase lipid metabolism in Leydig cells cannot be excluded. The question arises whether adipokines may modulate Leydig cell functioning through a mechanism that has not yet been defined. It is worth adding that our previous morphometric analysis [40] revealed a marked increase of interstitial volume, accompanied by a decreased number of Leydig cells per 1 mm^2^ of the interstitial space, indicating the occurrence of Leydig cell hypertrophy after flutamide exposure. Elucidation of whether a compensatory mechanism within Leydig cells exists will require further study at the ultrastructural level.

Interestingly, investigating the efficacy of flutamide, we found specific effects that occurred within Leydig cells. Levels of nectin-2 and afadin were down-regulated, mainly in the interstitium and, to a lesser extent, within seminiferous tubules. Decreased expression of junction proteins following flutamide exposure mirrored a report by Fiorini et al. [44], which showed that a gap junction protein (connexin43; Cx43) was a specific target for testicular toxicants. According to Takai and Nakanishi the presence of the nectin-afadin signals in testes reflects their role in the stability of cell to cell contacts [66]. In mice with *nectin-2* deficiency, malformations in spermiogenesis, impaired sperm morphogenesis and male-specific infertility have been observed [67,68]. An involvement of the nectin-afadin complex in other heterotypic junctions between different testicular cell types, i.e., Sertoli cell-spermatid junctions, has recently been reported [69]. As shown in our study, nectin-2 and afadin immunosignals were also observed in the apical part of seminiferous tubules; however, both protein stainings were lower in intensity compared to those in the interstitial compartment of the testis. We assume that changes in the expression levels of cell–cell junction proteins in Leydig cells may lead to the impairment of para- and autocrine communication, important for adipokines’ functioning. This assumption is in accordance with previously published reports showing that the adipokines function as paracrine and/or autocrine factors in the female reproductive tract (for review, see [4,12,70]).

**Figure 6:**
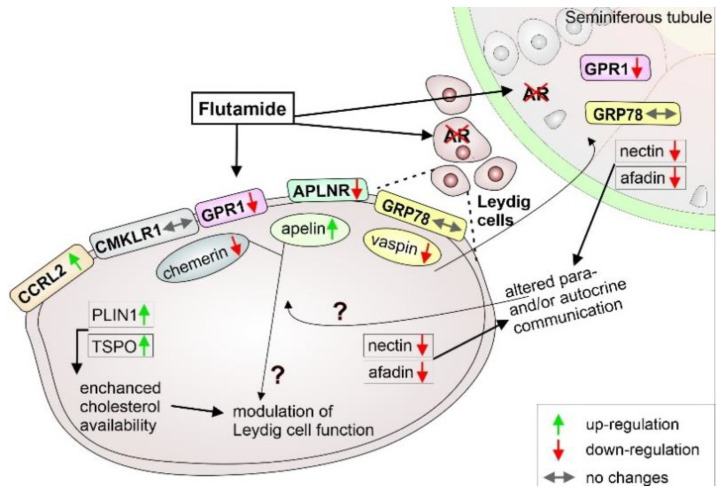
Schematic representation of local effects of flutamide in the rat testis. Interactions of flutamide with the adipokines, their receptors, the genes involved in lipid metabolism and the proposed relationship with the adherens junction proteins are presented. Flutamide, by blocking the androgen receptor (AR) in testicular cells, induces alterations in chemerin, apelin and vaspin gene expression levels, mainly in Leydig cells. Distribution of CMKLR1, CCRL2 and APLNR, restricted only to Leydig cells, indicates autocrine action of chemerin and apelin, whereas the localisation of GPR1 and GRP78 within Leydig cells and seminiferous tubule indicates the possibility of para- and/or autocrine action of chemerin and vaspin. Both chemerin and apelin, acting through their own receptors, are implicated in modulation of metabolic activity of Leydig cells by increasing the gene expression of lipid metabolism-modulating proteins, PLIN1 and TSPO. Alterations in adherens junction protein expression presumably lead to disturbed local communication, important for proper functioning of adipokines. Additionally, vaspin acting through GRP78 likely regulates Sertoli and germ cell functioning. Hypothetical interactions are marked with “?”.

Altogether, the results presented here indicate that androgen deprivation by flutamide influences the functioning of chemerin, apelin and vaspin in testes of adult rats and alters the gene expression of PLIN1 and TSPO, enhancing cholesterol availability in Leydig cells. Alterations in adherens junction protein expression in the testis confirm the flutamide efficacy and presumably may result in impaired para- and autocrine communication, influencing adipokines’ functioning. Figure 6 presents a schematic illustration of the interactions of flutamide with the adipokines, their receptors, the genes involved in lipid metabolism and the relationship with the adherens junction proteins. We realise that suggested interactions are speculative and should be tested in a future study. However, the distribution of immunoreactive chemerin, apelin, and vaspin and their own receptors showed here adds a new information about the adipokine cellular targets within rat testis.

## 4. Materials and Methods

### 4.1. Animals and Tissue Preparation

Adult Wistar male rats from five different litters were distributed by random allocation into experimental (*n* = 6) and control (*n* = 6) groups. Rats in the experimental group received a daily subcutaneous injection of flutamide (2-methyl-N-[4-nitro-3-(trifluoromethyl)-phenyl]propamide; (cat. no.: F9397, Sigma-Aldrich, St. Louis, MO, USA) suspended in corn oil for seven consecutive days (each dose, 50 mg⁄ kg body weight). Control rats were injected with corn oil only. A dose of flutamide was selected as high enough to produce changes in the expression of cell–cell junction protein levels without generating testicular germ cell loss from the seminiferous tubules. Treatment protocol was evaluated according to the literature and our previously published data [40,45,71]. The animals were housed in a fully controlled standard environment (temperature 22 °C, relative humidity 55% ± 5%, light:dark cycle of 12:12 h), with access to water and food ad libitum (Agropol, Motycz, Poland). The animals from both groups were sacrificed by cervical dislocation after anesthesia with 4–5% (*v*/*v*) isoflurane at 90 days of age. Testes were dissected and blood samples were collected from the dorsal aorta using a heparinised syringe. Plasma concentrations of adipokines, cholesterol and testosterone were measured using commercially available ELISA kits. Fresh tissue fragments were snap-frozen and stored at −80 °C for qRT-PCR and WB analyses, or fixed in 4% paraformaldehyde, and embedded in paraplast for IHC [72].

The use of animals and all protocols of animal experiments were approved by the Local Ethics Committee in Krakow (permission numbers: 116/2012 and 85/2019).

### 4.2. Immunohistochemistry

For immunohistochemistry 5 μm thick paraffin-embedded sections of testes were used. For improved IHC staining antigen retrieval, non-specific protein blocking and endogenous peroxidase blocking were carried out, as described previously [40]. Briefly, testes sections were submerged in 10 mM citrate buffer (pH 6.0) and/or in Tris-EDTA buffer (pH 9.0) and boiled in a microwave oven (2–3 min, 750 W). Subsequently, the sections were incubated (overnight at 4 °C) with primary antibodies against chemerin, CCRL2, CMKLR1, GPR1, apelin, APLNR, vaspin and GRP78, PLIN1 and TSPO as well as nectin-2 and afadin proteins (for details see Table 2).

Thereafter, sections were incubated for 30 min with biotinylated secondary antibody goat anti-rabbit, horse anti-mouse or horse anti-goat (1:400; cat. no.: BA-1000, cat. no.: BA-2000 or cat. no.: BA-9500, respectively; Vector, Burlingame, CA, USA). All antibodies were diluted in Tris-buffered saline (TBS; 0.05 M Tris-HCl, 0.15 M NaCl, pH 7.6). During procedure, after each incubation, sections were washed in TBS 3 times for 5 min each. The staining signal was amplified by incubation for 30 min with avidin-biotinylated horseradish peroxidase complex (ABC/HRP; 1:100; Vectastain Elite ABC Reagent, Vector Lab.) and by 0.05% 3.3′-diaminobenzidine tetrachloride (DAB; cat. no.: D5637, Sigma–Aldrich) dissolved in TBS containing 0.01% (*v*/*v*) H_2_O_2_ and 0.07% (wt/v) imidazole. Lastly, all sections were rinsed and additionally stained with Mayer’s haematoxylin (cat. no.: 124687402, CHEMPUR, Piekary Slaskie, Poland), dehydrated and coverslipped with DPX mounting medium (Sigma-Aldrich). All procedures were performed identically at the same time to ensure uniformity and specificity of the staining [38]. As a negative control, primary antibody was omitted and replaced by an irrelevant IgG. Sections were examined with a Leica DMR microscope (Leica Microsystems GmBH, Wetzlar, Wetzlar, Germany).

For quantitative analysis, digital color images of testes sections were acquired using a charge-coupled device (CCD) video camera mounted on an optical microscope (Microphot, Nikon, Japan). Images were processed using ImageJ software (National Institutes of Health, Bethesda, MD, USA). The intensities of IHC staining were expressed as relative optical density (ROD) of diaminobenzidine brown reaction products, defined previously by Smolen [73]. Image analysis were performed on 24 randomly selected tissue sections of control (*n* = 6) and flutamide-treated (*n* = 6) testes. Measurement values were expressed as mean ± SD.

### 4.3. Western Blot Analysis

Tissue samples of the testes were homogenised in cold-ice radio-immuno-precipitation assay buffer (RIPA, pH 8.0; Thermo Scientific; Inc., Rocheford, Il, USA) containing protease inhibitor cocktail (Sigma-Aldrich), sonicated and centrifuged as described previously [74]. Protein concentrations were determined by performing the Lowry assay (Bio-Rad Labs, GmbH, München, Germany). Proteins were separated by sodium dodecyl sulphate polyacrylamide gel electrophoresis (SDS-PAGE) and immobilised in polyvinylidene fluoride membranes, as described before [67]. To prevent non-specific binding of antibodies membranes were raised with non-fat dry milk (5%, wt/v) containing 0.1% Tween 20 (*v*/*v*). After blocking, the membrane was incubated with the primary antibodies (Table 2) at 4 °C overnight. Next, after washing membranes with TBST buffer (0.05 M Tris-HCl, 0.15 M NaCl, pH 7.6 containing 0.1% Tween 20) secondary antibody was added (1:3000; Vector Laboratories, Burlingame, CA, USA), and incubated for 1 h at room temperature with gentle rocking. Chemiluminescent signals on Western blot were detected and captured with a ChemiDocTM XRS + System (Bio-Rad Laboratories). All blots were stripped at 50 °C for 30 min (stripping solution, 62.5 mM Tris- HCL, 100 mM 2-mercaptoethanol, 2% SDS (wt/v) (pH 6.7)), and subsequently re-probed with an additional antibody against β-actin (dilution, 1:3000; Sigma-Aldrich), which was used as a loading control and for normalisation. Protein molecular weight marker (ColorBurst Electrophoresis Marker, Sigma-Aldrich) was run, to calculate molecular weight of target proteins. The bands were analysed and quantified with Image LabTM 2.0 (Bio-Rad Laboratories).

### 4.4. RNA Extraction, Reverse Transcription

The frozen testes were used for RNA isolation with TRIzol reagent (Life Technologies, Gaithersburg, MD, USA). To remove DNA contamination from RNA samples TURBO DNase free Kit (Ambion, Austin, TX, USA) was applied, following the manufacturer’s protocols. For assessing RNA quality the A_260_:A_280_ ratio was measured using a NanoDrop ND2000 Spectrophotometer (Thermo Scientific, Wilmington, DE, USA) and by electrophoresis. The purified total RNA (1 μg) was reverse transcribed into cDNA using cDNA reverse transcription kit (Applied Biosystems, Carlsbad, CA, USA) according to the manufacturer’s instructions. The total mixture (20 μL) contained cDNA, random primers, dNTPmix, RNAse inhibitor and reverse transcriptase (RT). For each RNA sample the same reactions in the absence of RT were performed.

### 4.5. Real-Time Quantitative RT-PCR

Real-time RT-PCR analyses were carried out using a StepOne Real-Time PCR system (Applied Biosystems) with the cDNA templates and optimised standard conditions as described previously [75]. The primers for the genes, provided by Institute of Biochemistry and Biophysics, Polish Academy of Sciences (Warsaw, Poland), are listed in Table 3.

Amplification efficiency (ranging from 97% to 104%) was calculated as described by Svec et al. [76]. The amplification was performed with 10 ng cDNA, 0.5 mM of each primers and SYBR Green master mix (Applied Biosystems). The amplification specificity of expected PCR products was confirmed by melting curve analysis and agarose gel electrophoresis. All experiments were performed in triplicate and repeated in independent experiments three times. The 2^-ΔΔCt^ method was used to determine the relative quantification (RQ) of the target genes: *Chemerin*, *Ccrl2*, *Cmklr1*, *Gpr1*, *Apelin*, *Aplnr*, *Vaspin*, *Grp78, Nectin2, Afadin*, *Plin1* and *Tspo*. The mRNA expressions were normalised to the mean expression of reference genes *β-actin*, *Gapdh* and *Rpl13a* (RQ = 1) [77]. In Figure 3, Figure 4 and Figure 5, as an internal control, representative *β-actin* transcript level was shown. In all real-time RT-PCR reactions, negative controls were carried out.

### 4.6. Enzyme-Linked Immunosorbent Assay (ELISA) Analysis

Commercially available rat enzyme-linked immunosorbent assays (ELISA) were used to quantify total chemerin (cat.no.: MBS774364, MyBioSource Inc., San Diego, CA, USA), apelin (cat. no.: MBS725297, MyBioSource Inc.), vaspin (cat. no.: MBS2501454, MyBioSource Inc.), cholesterol (cat. no.: E0784Ra, Bioassay Technology Laboratory) and testosterone (cat. no.: EIA-1559, DRG, Inc. Int. Springfield, NJ, USA) concentrations in plasma. For all measurements, samples of control and flutamide-treated rats were used according to manufacturer’s protocols. Samples were run in triplicate within the same experiment and measured by using a microplate reader (Labtech LT-4500, Pocklington, UK).

### 4.7. Statistical Data Analysis

The raw data were processed using Statistica 10 software (StatSoft Inc., Tulsa, OK, USA). Normality of data distribution was tested using the Shapiro–Wilk W-test. The non-parametric Mann–Whitney U-test was conducted to determine statistical differences in protein and mRNA expression levels. Data were presented as mean ± SD and considered statistically significant at * *p* < 0.05, ** *p* < 0.01, *** *p* < 0.001.

## 5. Conclusions

The presence of chemerin, apelin and vaspin and their own receptors demonstrated here provides new information about the adipokine cellular targets within rat testes. Furthermore, our results indicate that androgen signalling disrupted by flutamide influences the functioning of abovementioned adipokines and alters the gene expression of PLIN1 and TSPO, enhancing cholesterol availability in Leydig cells. Alterations in *Nectin2* and *Afadin* gene expression levels confirm the flutamide efficacy to induce specific effects within Leydig cells and presumably may lead to impaired para- and autocrine communication, crucial for proper functioning of adipokines.

## Figures and Tables

**Table 1:** Serum concentrations of studied adipokines in control and flutamide-treated rats.

	Control	Flutamide
Chemerin	12.736 ± 0.650 ng/mL	13.346 ± 1.116 ng/mL
Apelin	176.386 ± 41.236 pg/mL	192.110 ± 67.429 pg/mL
Vaspin	452.349 ± 15.379 pg/mL	488.864 ± 36.916 pg/mL

Data are expressed as means ± SD.

**Table 2:** Details of primary antibodies used for Western blot (WB) and immunohistochemistry (IHC).

Antibody	Host Species	Vendor	Cat. Number	Dilution
Anti-chemerin	Rabbit	Abcam	ab103153	1:500 (WB) 1:200 (IHC)
Anti-CCRL2	Rabbit	Sigma-Aldrich	SAB2100371	1:1000 (WB) 1:200 (IHC)
Anti-CKMLR1	Rabbit	Abcam	ab64881	1:500 (WB) 1:200 (IHC)
Anti-GPR1	Goat	Santa Cruz Biotechnology	sc-48178	1:200 (WB) 1:200 (IHC)
Anti-apelin	Mouse	Santa Cruz Biotechnology	sc-293441	1:1000 (WB) 1:200 (IHC)
Anti-APLNR	Mouse	Santa Cruz Biotechnology	sc-73713	1:500 (WB) 1:200 (IHC)
Anti-vaspin	Rabbit	ThermoFisher Scientific	PA5-30989	1:500 (WB) 1:500 (IHC)
Anti-GRP78	Rabbit	ThermoFisher Scientific	PA5-19503	1:500 (WB) 1:500 (IHC)
Anti-PLIN1	Goat	Abcam	ab61682	1:300 (WB) 1:400 (IHC)
Anti-TSPO	Rabbit	Bioassay Technology Laboratory	BT-AP06962	1:400 (WB) 1:200 (IHC)
Anti-nectin-2	Rabbit	Bioassay Technology Laboratory	BT-AP04642	1:1000 (WB) 1:100 (IHC)
Anti-afadin	Rabbit	Bioassay Technology Laboratory	BT-AP06988	1:500 (WB) 1:100 (IHC)
Anti-β-actin	Mouse	Sigma-Aldrich	A2228	1:500 (WB)

**Table 3:** Sequences of forward and reverse primers.

Genes	Primers (5’–3´)	Product Size (bp)	Annealing Temp. (°C)
*Chemerin*	5′-TGTGCAGTGGGCCTTCCA-3′ 5′-CAAAGGTGCCAGCTGAGAAGA-3′	71	55
*Ccrl2*	5′-TGTGTTTCCTGCTTCCCCTG-3′ 5′-CGAGGAGTGGAGTCCGACAA-3′	101	60
*Ckmlr1*	5′-CAAGCAAACAGCCACTACCA-3′ 5′-TAGATGCCGGAGTCGTTGTAA-3′	234	52
*Gpr1*	5′-GGAGCTCAGCATTCATCACA-3′ 5′-GACAGGCTCTTGGTTTCAGC-3′	243	53
*Apelin*	5′-AGACCCCGGAGGCTAAGGAGTT-3′ 5′-TCCGTCATAGTGTCCTCCATCA-3′	300	62
*Aplnr*	5′-GGACTCCGAATTCCCTTCTC-3′ 5′-CTTGTGCAAGGTCAACCTCA-3′	140	53
*Vaspin*	5′-CTGAAACTGGCTAAGAACT-3′ 5′-CACCTGCCTTGAAAGTAAAT-3′	191	47
*Grp78*	5′-CTGGGTACATTTGATCTGACTGG-3′ 5′-GCATCCTGGTGGCTTTCCAGCCATTC-3′	398	64
*Plin1*	5′-TGCGCAAGAAGAGCTGAGTA-3′ 5′-AGAGGCCAACCTGAAGGAGT-3′	128	53
*Tspo*	5′-GCTGGACACTCGCTCCCA-3′ 5′-CATACCCCATGGCCGAATAC-3′	51	54
*Nectin2*	5′-CGGAACTGTCACTGTCACCA-3′ 5′-GACACTTCAGGAGGGTAGCG-3′	150	57
*Afadin*	5′-CCGACATCATCCACCACTGG-3′ 5′-CAGCATTCGCATATCAGGTCG-3′	218	60
*β-actin*	5′-AAGTACCCCATTGAACACGG-3′ 5′-ATCACAATGCCAGTGGTACG-3′	257	52
*Gapdh*	5′-ACCACAGTCCATGCCATCAC-3′ 5′-TCCACCACCCTGTTGCTGTA-3′	452	55
*Rpl13a*	5′-CATTCGAACGTCTGCCCTAT-3′ 5′-GTTTCTCAGGCTCCCTCTCC-3′	128	56

## Supplementary Materials

The following are available online at https://www.mdpi.com/1422-0067/21/12/4439/s1, Original full Western blots corresponding to cropped lanes shown in Figure 2, Figure 4B and Figure 5B.
