# Supplementary material for: Flutamide Alters the Expression of Chemerin, Apelin, and Vaspin and Their Respective Receptors in the Testes of Adult Rats
Source: Int J Mol Sci. 2020 Jun 22;21(12):4439. doi: 10.3390/ijms21124439 (PMC7378763; doi:10.3390/ijms21124439)
Supplement: Supplementary file 1 [file ijms-21-04439-s001.pdf]

Figure 2

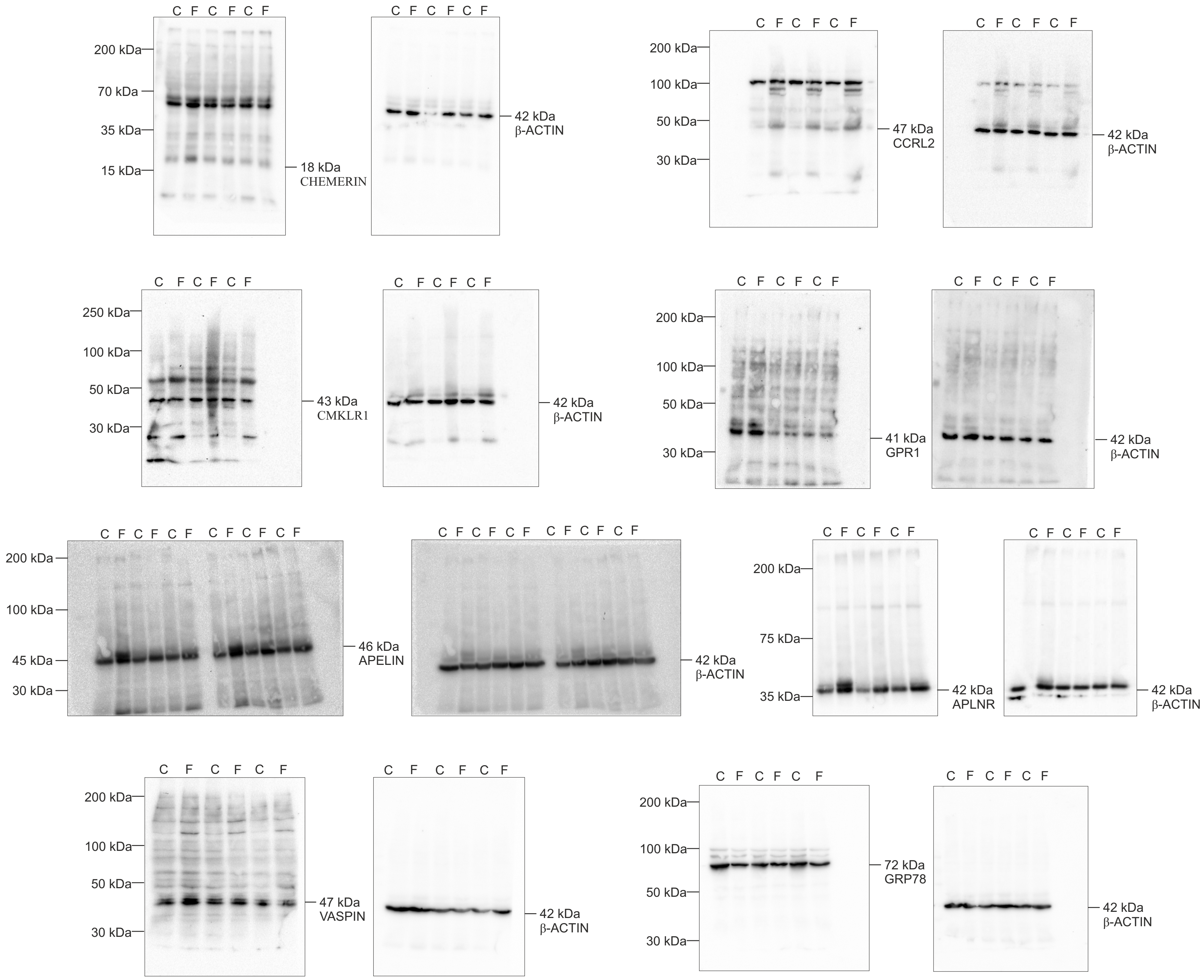

Figure 4B

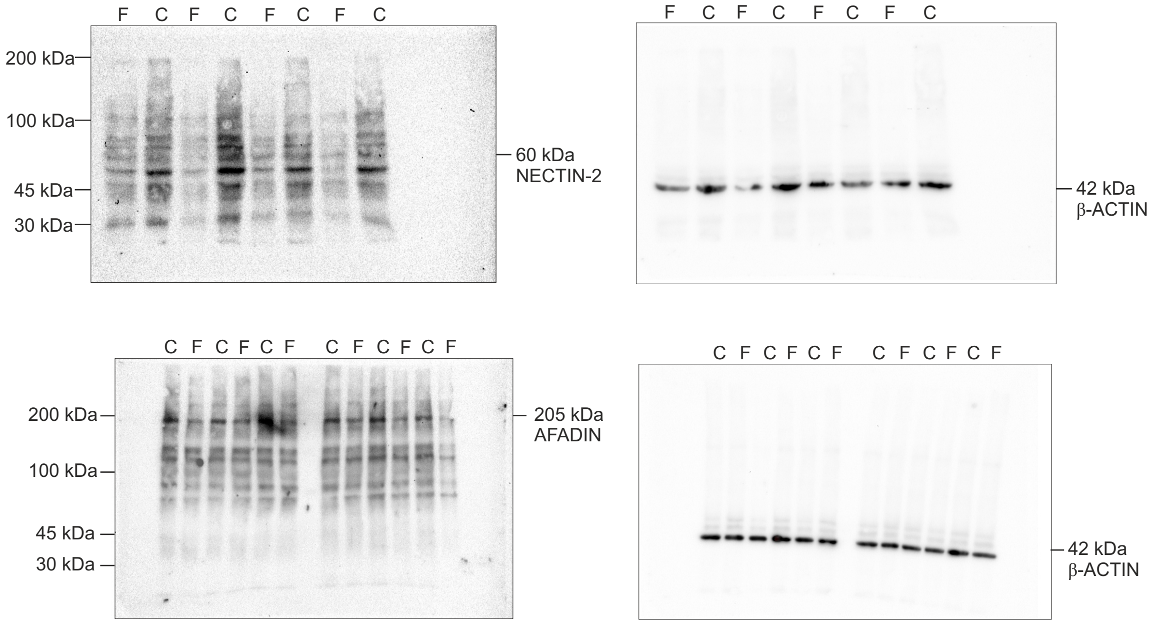

Figure 5B

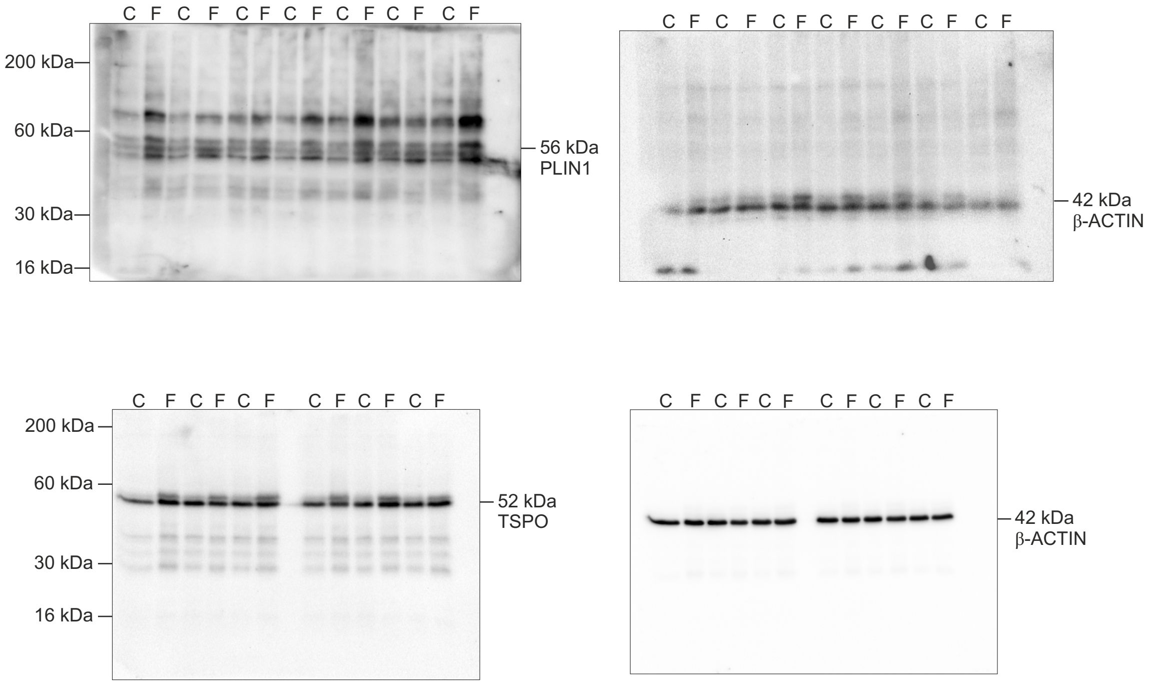

Uncropped images of all Western blots. The relative position of the protein band corresponding to the ColorBurst Electrophoresis Marker (Sigma-Aldrich) is noted to the left. C: control; F: flutamide
